# Servicemen’s features of professional reliability

**DOI:** 10.1192/j.eurpsy.2021.1183

**Published:** 2021-08-13

**Authors:** Y. Novikova, D. Boyarinov, L. Gubaidulina, A. Kachina, V. Barabanshchikova

**Affiliations:** Faculty Of Psychology, Lomonosov Moscow State University, Moscow, Russian Federation

**Keywords:** Professional Reliability, Servicemen, Chronic Stress, Personal Characteristics

## Abstract

**Introduction:**

In our days the professional reliability of servicemen is an important issue. Servicemen not only protect the state but also ensure a stable and harmonious life of society (Vagin, 2012). This profession is extreme for life, therefore the significance of human error in this profession is very high. Study of personal characteristics forming professional reliability is important for professional psychological selection. The study was supported by the RFBR #19-013-00799 А.

**Objectives:**

Study of the psychological factors for reliability of servicemen.

**Methods:**

The study involved 708 servicemen, the average age of 20.3 (min – 18, max – 32), the sample consisted only of men. The methodological package included the following methods: Managerial stress survey — MSS (Leonova, 2007), The Sixteen Personality Factor Questionnaire (Kapustina, 2001).

**Results:**

The exploratory factor analysis revealed that the professional reliability includes the following personal characteristics: low level of Сhronic stress (-0.851), Emotional Stability (0.823), Motivational Distortion (0.761), Apprehensiveness (-0.716). The Kaiser-Meyer-Olkin Measure of Sampling Adequacy = 0.781, and Bartlett’s test of sphericity = 865.26 (p = 0.0001), the total variance of this factor is 62.4%.

**Conclusions:**

According to this factor, the profile of professional reliability of each serviceman was compiled: low stress level, self-confidence, non-fearfulness, efficiency, high self-esteem, personal maturity. In the future, confirmatory factor analysis will be performed, and the relationship of this scale with objective data will be investigated.

